# About Face: Is Virtual Group Delivery of Clinical Emotional Freedom Techniques (EFT) as Effective as Face-to-Face Group Delivery in Improving Psychological and Physiological Markers of Health?

**DOI:** 10.3390/healthcare14060784

**Published:** 2026-03-20

**Authors:** Elizabeth Boath, Dawson Church, Peta Stapleton

**Affiliations:** 1 Department of Social Work and Social Welfare, School of Health and Social Care, University of Staffordshire, Stoke-on-Trent ST4 2DE, UK; 2 National Institute for Integrative Healthcare, Petaluma, CA 94953, USA; 3 School of Psychology, Bond University, Robina, QLD 4226, Australia

**Keywords:** emotional freedom techniques, psychotherapy, telemedicine, anxiety disorders, depressive disorder, treatment outcomes

## Abstract

**Introduction:** Over 100 studies demonstrate the efficacy of Emotional Freedom Techniques (EFT), an evidence-based therapeutic method. However, most research is on in-person delivery of EFT. Only a few studies examine EFT delivered virtually, and to date no research has provided a direct comparison of group virtual EFT to group in-person delivery. **Objectives:** Delivery of EFT shifted to online platforms in the wake of the 2020 COVID-19 pandemic. This makes a comparison of virtual delivery to in-person delivery timely. The research question of whether online group delivery is as effective as in-person group delivery is of high clinical relevance, given the increased access and convenience offered by virtual treatment options. **Methods:** Participants in the online group were a convenience sample of 172 participants drawn from four four-day virtual EFT training sessions. Changes in psychological and physiological symptoms were measured pre, post, and at six-month follow-up using the Patient Health Questionnaire (PHQ-4). The two-item Post Traumatic Stress Disorder (PTSD) Checklist (PCL), the Happiness Scale, and the QuickDASH pain scale. These results were then compared to those of a previously published study of in-person group EFT (*n* = 203) that used an identical training curriculum delivered face-to-face. Due to COVID restrictions, the physiological measures used in the face-to-face delivery could not be replicated in the virtual group. **Results:** Online group EFT demonstrated significant improvements in PTSD, anxiety, depression, pain, and happiness (all *p* < 0.001) pre to post EFT. These improvements were maintained at six-month follow-up for PTSD (*p* < 0.001), depression (*p* = 0.048), pain (*p* = 0.002), and happiness (*p* < 0.001). Although there was a reduction in anxiety in the online group at six-month follow-up, this did not reach significance (*p* = 0.102). When compared to the in-person group (pre-COVID), the percent change in symptoms, while still clinically and statistically significant, was for most conditions smaller in the virtual group (post COVID) at both post and follow-up time points. **Conclusions:** EFT is associated with significant improvements in psychological and physiological conditions including PTSD, anxiety, depression, pain, and happiness, whether delivered virtually in groups or in-person in groups. The psychological and physiological benefits identified in online treatment are similar to those found during in-person delivery, though not as large or clinically significant. This finding is consistent with the literature demonstrating that online treatment is an effective method of delivering psychological therapies. The results reinforce other studies showing COVID produced a significant increase in mental health symptoms. Published treatment guidelines already recommend in-person EFT as an efficient and potentially cost-effective first-line intervention in primary care; virtual group EFT can be similarly recommended.

## 1. Background

Over 100 studies, including over 50 randomized controlled trials, have shown Emotional Freedom Techniques (EFT) to be effective [1]. Randomized controlled trials have shown EFT to be effective for a range of psychological conditions including depression, anxiety, phobias, and post-traumatic stress disorder (PTSD) and physiological issues including pain, insomnia, and autoimmune disorders [1]. EFT has also been shown to enhance sports and professional performance and to reduce the biological markers of stress [1]. Meta-analyses have found moderate to large effect sizes for EFT [2,3,4,5,6,7]. Successful independent replication studies have also been carried out for studies of EFT for anxiety, depression, PTSD, phobias, sports performance, and cortisol levels [1,8]. Clinical guidelines for the use of EFT have been devised [9,10], and while these guidelines support the use of EFT in clinical practice, EFT can also be used as a self-help tool. Tens of millions of people are using EFT in this way worldwide [1,11].

However, most of these studies research in-person delivery of EFT and only a few studies examine EFT delivered virtually [1,12,13]. Furthermore, no studies to date provide a direct comparison of virtual to in-person delivery.

The research question of whether online delivery is as effective as in-person therapy is of high clinical relevance, given the increased access and convenience offered by virtual treatment and training options as well as the potential cost and efficiency savings associated with online delivery. Of additional value is determining whether the theoretical framework presented above carries forward into virtual treatment. A systematic review and meta-analysis of 54 RCTs found little to no difference in effectiveness for various mental health conditions, including anxiety and depression, between therapist-guided remote and in-person CBT [14]

In March 2020, the World Health Organization (WHO) declared a global pandemic resulting from coronavirus (COVID-19) [15]. As with other therapies, the delivery of EFT shifted almost overnight from face-to-face to online platforms in the wake of the COVID-19 global pandemic and the resulting “covid restrictions” [16]. This provided an unprecedented opportunity to compare and contrast the virtual delivery of EFT to in-person delivery.

In a prospective study, Bach and colleagues (2019) assessed the impact of in-person delivery of EFT on the psychological and physiological symptoms of anxiety, depression, PTSD, pain, cravings, and happiness [17]. They found significant reductions, all *p* < 0.001, in anxiety (−40%), depression (−35%), post-traumatic stress disorder (−32%), pain (−57%), and cravings (−74%) and a significant increase in happiness (+31%, *p* = 0.001). These gains were also maintained at six-month follow-up, indicating that EFT resulted in positive health effects and increased mental well-being.

The current prospective study aimed to replicate the Bach et al. (2019) study using a virtual presentation of EFT [17]. However, restrictions due to COVID-19 meant that the battery of physiological tests for blood pressure, heart rate, and cortisol that were included in the Bach study could not be carried out and so the relationship of psychological symptoms to these physiological markers could not be replicated. Furthermore, while the study by Bach used the Hospital Anxiety and Depression Scale [18,19] to assess anxiety and depression, use of the HADS has declined since the turn of the century as shorter and more sensitive instruments have become available, and its once wide acceptance has diminished [18]. The ubiquitous Patient Health Questionnaire [20] was therefore used instead in the virtual study. This precluded a direct statistical comparison so effect sizes and symptom change were used instead.

## 2. Methodology

The study procedures and assessments were reviewed by the research ethics committee of the National Institute for Integrative Healthcare [21] and were determined to present non-significant risk to participants (NIIH20200102). All participants provided informed consent. Participants in the four virtual workshops were assessed for anxiety and depression using the Patient Health Questionnaire [20]. The PHQ-4 includes four questions with each item scored from 0 to 3. The total score is determined by adding together the scores of each of the four items (total ranging from 0 to 12). Total scores of 0–2 are rated as normal, 3–5 as mild, 6–8 as moderate, and 9–12 as severe. A total score ≥ 3 for the first two questions suggests a likely diagnosis of anxiety while a total score ≥ 3 for the last two questions suggests a likely diagnosis of depression.

Participants in the original face-to-face study [17] were assessed for anxiety and depression using the Hospital Anxiety and Depression Scale [18,19]. HADS includes seven questions on depression and seven questions on anxiety. Items are scored from 0 to 3 and separate scores are calculated for anxiety and depression. A score of >8 for either anxiety or depression is considered clinically significant. However, as use of the HADS has declined, the PHQ has been used instead for subsequent EFT research. Both HADS (Cronbach’s alpha 0.83 for anxiety and 0.82 for depression) and PHQ (Cronbach’s alpha 0.80) demonstrate acceptable reliability and validity. Because scores on the two instruments are not interchangeable, we report the size of treatment effect in the form of paired Cohen’s d (standardized response mean) as well as percent change in symptoms.

Post-traumatic stress disorder (PTSD) was assessed with the two-item form of the PTSD Checklist [22]. Prior research has shown the two-item or six-item versions of the PCL to be effective PTSD screening instruments for primary care settings. We selected the two-item version, which has a Cronbach’s alpha 0.79, due to its brevity and the higher participant compliance rate for shorter assessments.

Happiness was assessed by the single item Happiness scale ([23]; test–retest reliability *r* = 0.74). Pain was measured using the visual analogue version of the QuickDASH ([24]; Cronbach’s alpha 0.92). 

Psychological testing was the same at all four virtual workshops, with pre and post measures completed by participants online and at a follow-up point six months later. Pre measures were completed prior to the first day of each workshop and post measures at the end of the last day. Data analysis was performed only for participants for whom both pre and post measures were available. The dropout rate at the end of the last day was 16.3% in the face-to-face group and 0% in the virtual group. The dropout rate at six-month follow-up was 58% in the face-to-face group and 46.5% in the virtual group.

## 3. Clinical EFT Intervention

All instructors in virtual EFT received training and held certification in Clinical EFT from EFT Universe [25]. The application of EFT was performed with fidelity to the third edition of The EFT Manual [26]. Twelve hours of the training was dedicated to Clinical EFT demonstrations, participant practice sessions, and feedback. The remaining 12 h was devoted to lectures about the physiology of stress, the 48 techniques, the nature and treatment of trauma, scope of practice, ethics, and the application of EFT to specific populations such as children, athletes, and employees. Clinical EFT was employed as a peer-to-peer coaching method, focusing on alleviating the severity of emotional distress rather than providing diagnosis or mental health treatment. The Zoom platform was employed, and participants were frequently placed in “breakout rooms” to facilitate individual practice. Technical issues were addressed by having participants log on a few minutes early for a video and audio check, while an assistant was available for any who experienced technical challenges during the workshops. Fidelity was maintained by using the same Powerpoint presentations for all workshops, which include coverage of all 48 Clinical EFT techniques.

The “Borrowing Benefits” group delivery protocol for EFT, outlined in The EFT Manual [26], was utilized during the virtual sessions as in the earlier in-person study [17]. The term “Borrowing Benefits” derives from the practice of members of a group self-applying EFT while observing practical demonstrations. It can be used either virtually or in person. Previous research has shown Borrowing Benefits to be associated with simultaneous reductions in symptoms of PTSD, anxiety, and depression [9,17], as well as with reductions in cortisol [13].

## 4. Results

Post-hoc power analysis using observed effect sizes and sample sizes indicates very high power (>99%) for detecting within-group changes in both the virtual (*d* = 0.62, *n* = 172) and face-to-face (*d* = 0.68, *n* = 170) cohorts. For a small between-group effect (*d* = 0.20), achieved power is approximately 45%, which is modest. Thus, the study was well-powered for detecting pre–post improvements within each modality, but less so for small differences between modalities.

The size of treatment effect was measured using Cohen’s *d*. Paired Cohen’s *d* was computed as *d*(z) = mean (Post–Pre)/SD (Post–Pre) with bootstrap 95% confidence intervals. For outcomes where higher scores indicate improvement (Happiness) we report *d* as-is; for outcomes where lower scores indicate improvement (Pain, PTSD, Anxiety, Depression) we report the absolute value and note that improvements correspond to score reductions.

Immediate and 6-month follow-up changes were summarized as paired Cohen’s *d* with 95% bootstrap CIs for each outcome and instrument. Anxiety/depression effects for the face-to-face cohort are reported using HADS while virtual cohort effects are reported using PHQ.

Table 1 shows the baseline characteristics of the study participants at recruitment for the face-to-face and virtual delivery. There was no significant difference in gender (*p* = 0.435) between the two groups and the majority of participants in both groups were women. However, there was a significant difference in age (*p* = 0.004), with the virtual group having a slightly younger mean age.

The majority of participants in both groups had a university education, including up to postgraduate level.

The mean scores for happiness, pain, and PTSD were statistically significantly different between the two cohorts at the outset. The participants across both the face-to-face and virtual delivery came from a total of 16 different countries, with most participants in each cohort coming from the USA, Canada, and the UK. Due to the use of the HADS in the face-to-face group and the PHQ in the virtual group, no statistical comparison of depression or anxiety could be carried out, though in the series of tables displayed below, we present the *p*-values and percent change in both groups side by side.

Statistical analyses were performed using JASP (an open-source program for statistical analysis; https://jasp-stats.org). No data were normally distributed and so participant scores for PTSD, happiness, anxiety, depression, and pain were compared before and after EFT using the Wilcoxon signed-rank test for paired samples. Table 2 shows the change in scores on each of the scales used pre and post the virtual Clinical EFT training.

Between the pre- and post-test time points, participants experienced highly significant decreases in anxiety, depression, PTSD, and pain and a highly significant increase in happiness (see Table 2). These significant participant gains were maintained at six-month follow-up for PTSD, happiness, depression, and pain but not for anxiety (See Table 3).

Table 4 provides a comparison of face-to-face versus virtual EFT from pre- to post-intervention. Both cohorts show highly significant changes following the intervention across all outcome measures.

Table 5 provides a comparison of face-to-face versus virtual EFT from pre to six-month follow-up. Both cohorts still showed significant changes six months after the intervention for depression, PTSD, and pain. Happiness in the face-to-face cohort did not reach significance, although it showed a 6% improvement. Anxiety in the virtual group showed a 12% reduction but did not reach significance.

Table 6 provides a comparison of face-to-face versus virtual EFT pre, post, and at six-month follow-up. There were significant differences between the cohorts for PTSD, pain, and happiness pre-EFT and significant differences between the two cohorts in PTSD and happiness post EFT. However, by the six-month follow-up, there were no significant differences in PTSD, happiness, or pain between the two groups.

Table 4, Table 5 and Table 6 provide a basis for comparing the results between the in-person and virtual groups. Generally, both treatment delivery methods produced statistically significant changes from pre to post and from pre to follow-up. In addition, for almost all conditions, the percent change in symptoms, an indication of clinical significance, was high in the virtual group, but even greater in the in-person group. Although HADS and PHQ are not directly comparable, the percentage change for these was also higher in the face-to-face group.

There was a significant difference between the two groups in pain at pretest (*p* < 0.001), with the face-to-face group having a higher mean pain. However, there was no significant difference between the two groups in the degree of pain reduction postintervention (*p* = 0.096) or at the six-month follow-up (*p* = 0.185), indicating a statistically similar treatment effect whether EFT was delivered virtually or in person.

There was a significant difference between the two groups in happiness at pretest (*p* < 0.001), with the face-to-face group having a higher mean happiness score. This difference was maintained postintervention (*p* < 0.001), with happiness being the only condition in which the virtual group demonstrated greater percent change than the in-person group. However, there was no significant statistical difference in happiness between the two cohorts at the six-month follow-up (*p* = 0.069), indicating statistically similar improvement in symptoms.

There was a significant difference between the two groups in PTSD symptoms pre-test (*p* = 0.012), with the face-to-face group having a higher mean PTSD score. There was a significant difference between the virtual and face-to-face groups in PTSD symptoms post EFT (*p* < 0.001) but not at the six-month follow-up (*p* = 0.826), indicating a statistically similar long-term treatment effect whether EFT was delivered virtually or in person.

**Figure 1:**
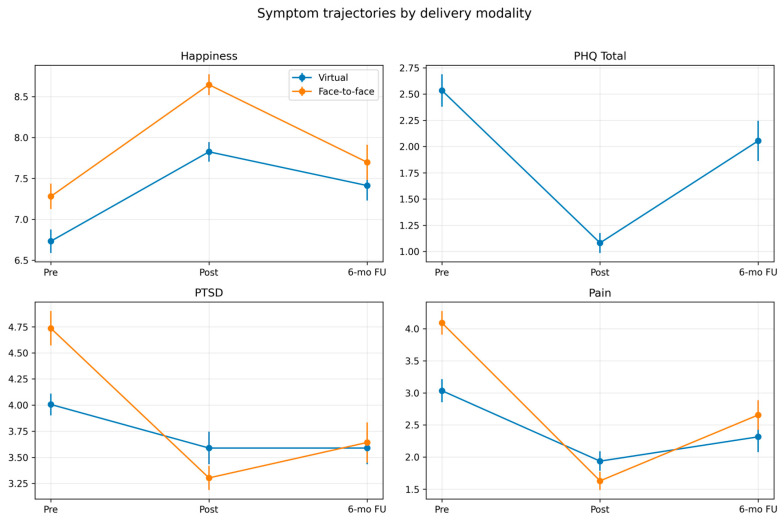
Symptom trajectories by delivery modality.

**Figure 2:**
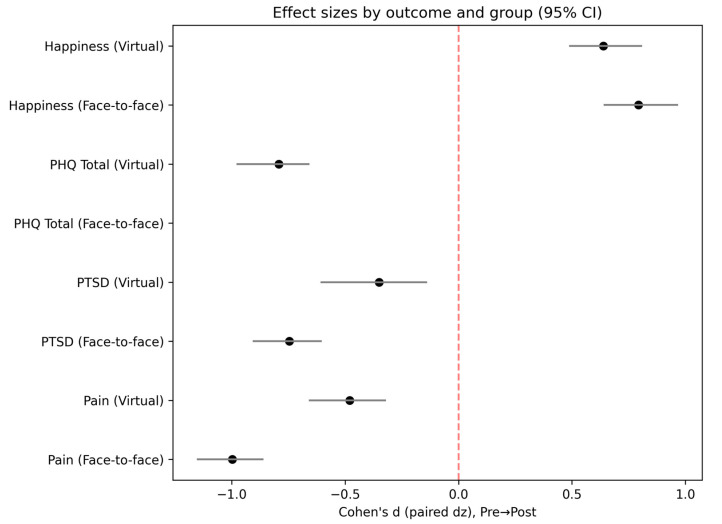
Effect sizes by outcome and group.

The country of residence data was available for the virtual group. Participants were predominantly drawn from the USA (124/173; 71.7%), Canada (20/173; 11.6%), and the UK (9/173; 5.2%); other countries each contributed ≤2%. Figure 1 and Figure 2 below graphically portray the trajectory of symptoms by group and condition, as well as effect sizes.

## 5. Discussion

The virtual delivery of EFT in this study stemmed from the sudden enforced move to online therapy triggered by the COVID-19 pandemic. Although some pre-pandemic research had demonstrated that online therapy can be as effective as in-person [27,28], many therapists still avoided online therapy due to concerns regarding its impact on the therapeutic alliance [29,30,31]. The pandemic, however, forced therapists to move their work online and offered an unprecedented opportunity to compare face-to-face group EFT with virtual group EFT. This study adds to the growing empirical evidence that virtual group therapy is feasible as well as having efficacy that approaches that of in-person group therapy. Online treatment is also accessible and acceptable to clients [32]. This study also supports the findings of Marmarosh et al. (2022) who found that group psychotherapy treatment options were effective at reducing depression, anxiety, and stress [33]. Social learning theory posits that individuals learn new behaviors by observing others, and in virtual environments and digital communities, this process can actually be amplified [34]. The immersive nature of these environments can foster higher social presence and engagement than traditional methods, leading to the effective acquisition of complex knowledge.

Although no physiological tests for blood pressure, heart rate, and cortisol that were included in the study by Bach et al. (2019) could be taken for comparison due to COVID-19 restrictions, the psychological results of the virtual group were compared to those of a previously published study of in-person group EFT (*n* = 203; [17]). Both delivery methods used an identical EFT curriculum.

Our results show that both populations evinced statistically significant symptom declines and increases in happiness between pretest and posttest and six-month follow-up time points. Symptoms of anxiety, depression, PTSD, and pain declined significantly in both the virtual and the face-to-face groups from pre to post. Statistically significant response rates were observed in both the virtual and face-to-face groups at six-month follow-up. Happiness significantly increased from pre- to post-test in both groups, however, this did not reach the level of statistical significance in the face-to-face group at six-month follow-up but did in the virtual group. However, this should be contextualized by the fact that in-person data were collected pre-COVID and virtual data post-COVID. Research on well-being and COVID-19 revealed that happiness decreased during the pandemic [35], while negative emotions increased [36,37]. This is likely to have had an impact on the results in the form of lower percent changes.

A 2022 meta-analysis of 20 studies conducted before COVID that compared virtual with in-person psychotherapy found that the results of the two delivery modalities did not differ significantly [38]. This suggests the possibility that the more modest improvements we found in virtual treatment post-COVID might have been influenced by the pandemic. However, the use of Zoom’s chat feature has been found to reduce verbal interaction and hinder the development of interpersonal relationships [39]. Nonetheless, the theoretical underpinnings of EFT in the form of its anchoring of mental cues in lived physical experience, congruence with established therapies such as PE and CBT, and the observed regulation of the nervous system, are all likely to be engaged by virtual as well as in-person treatment. Guidelines are being developed for the migration of group therapy to virtual settings, recommending enhanced procedures for screening, confidentiality, privacy, and technical competence [40].

Despite the positive results, this study has several limitations. The response rate at the six-month follow-up assessment was 50% (85/170) for the virtual group and 53% (92/172) for the in-person group, which may have affected the results. Systematic reviews and meta-analyses of follow-ups generally indicate that response rates in this category do not skew results [41,42,43], while Kristman et al. (2004) suggest that follow-up rates of 50–80% are acceptable in the context of long-term follow-up epidemiological cohorts; the degree to which dropouts affected the current results, if at all, is unknown [44].

Most participants in both groups identified as women and so the results may not be generalizable to people who identify as other genders. The participants in both the virtual and face-to-face group all self-selected to train in EFT. There was a significant difference in age (*p* = 0.004), with the virtual group having a slightly younger mean age than the face-to-face group. Age has been shown to have an impact on people’s attitudes towards the use of technology, with younger people being viewed as more “tech savvy” [45]. This younger mean age could reflect the possibility that younger people felt more confident and competent in using online technology and so elected to join the online group. Future research could explore this and the technological competence and confidence of online participants.

Younger age groups have also been shown to have experienced higher levels of depressive and generalized anxiety symptoms than older adults during COVID-19 [46]. As the virtual data were collected during this time, replicating the virtual delivery without the influence of COVID-19 is strongly recommended.

Despite the positive results, this study has several limitations. There was no randomization, no control or wait-list group, and the move to virtual EFT was forced by COVID-19 restrictions. The former limitations are mitigated by the large number of randomized controlled trials of EFT, over 50 currently [1], that show similar results.

Another limitation includes reliance on very brief, subjective self-report psychological measures for both the virtual and the face-to-face group. These were initially selected by Bach et al. (2019) to facilitate completion and reduce the compliance burden on participants [17]. Future research could compare brief self-report scales with longer instruments. There was therefore no formal clinical diagnosis, and future research could benefit from this. Only three of the original five scales were replicated in the virtual group. HADS was used to assess anxiety and depression in the face-to-face group while the PHQ was used in the virtual group, so no statistical comparison of anxiety and depression could be carried out. Though use of the HADS is rare today [18], future replications should use it anyway to enable a statistical comparison between face-to-face and virtual groups.

COVID-19 has been shown to have an adverse effect on mental health, including an increase in the prevalence of anxiety [47] and depression [48]. Kan and colleagues also demonstrated that COVID-related anxiety was highest in Europe and America, where the majority of participants were from [47]. They also reported that COVID-related anxiety was higher in women, who formed the majority of participants in this study. The study results may have been influenced accordingly.

The research did not explore the views, attitudes, or perceptions of the therapists or the participants on delivering or participating in group EFT online. Future research could therefore benefit from the inclusion of qualitative data, exploring which aspects of clients and therapists’ experiences resulted from the unprecedented landscape of COVID-19, and which resulted from online therapy more generally [49]. Replication of our study outside the unusual circumstances of COVID-19 will allow this.

Indeed, the sudden, abrupt, and involuntary move to unfamiliar online group therapy, combined with the stress, isolation, and uncertainty of living through the evolving pandemic, may have meant that clients and therapists alike found online therapy distressing and a negative experience [50]. However, although this does not appear to be the case in this study, future research could explore the views of therapists and clients using the Unified Theory of Acceptance and Use of Technology Framework [29].

The pandemic and the resulting lockdowns have been shown to have considerable negative effects on mental health across the globe and so it was surprising that anxiety, depression, pain, and PTSD were all reduced in the virtual group, while happiness increased. Indeed, during lockdown, Brodeur and colleagues (2020) found a significant increase in Google searches for terms like loneliness, boredom, worry, and sadness [51].

A portion of the effects shown could also have been due to nonspecific factors that are present in any therapy, such as sympathetic attention and the therapists’ ability to build a therapeutic alliance in a face-to-face or virtual group setting. However, as both the in-person and the virtual study indicate consistent positive outcomes, with different EFT practitioners delivering Clinical EFT, this suggests that, in line with the findings of Palmer-Hoffman and Brooks (2011), the improvements observed in the groups cannot be attributable to the unique online or in-person skills of a specific therapist [52]. Future research should also randomize participants between virtual and face-to-face group EFT, using the same therapists.

EFT’s epigenetic effects in online group versus face-to-face EFT could be further explored with use of salivary gene assays, as were used in Maharaj (2016) and similar studies [53]. A larger battery of psychological assessments and a clinical diagnosis would amplify the understanding derived from the results, while a second longer-term follow-up data point, such as a year, would reveal whether participant gains were sustained over a longer time period.

Although not addressed directly in this study, virtual group EFT is potentially a more efficient and cost-effective method of delivery, saving participants and therapists time and money, and future research could usefully explore the economic benefits of online versus face-to-face delivery.

This research also has implications for the future of Clinical EFT training, as although there was an involuntary transition to online therapy during the pandemic, online therapy is undoubtedly here to stay. Exposure to and specific training in online methods such as building rapport and the therapeutic alliance online [49] and avoiding distraction, which has been shown to be increased online [54], is therefore essential.

A virtual replication of the original Bach et al. (2019) study without the limitations placed on this research by COVID-19 restrictions would be useful [17]. In future, a randomized controlled trial (RCT) specifically designed to compare face-to face with virtual delivery of EFT training should be considered. This should address issues to minimize bias and increase the validity and reliability of the results. Studies specifically designed to compare online with face-to face delivery are beginning to emerge, for example, Stapelton and colleagues (2025) or Lin and colleagues (2025) [55,56].

## 6. Conclusions

This study collected data on the most common mental health conditions, such as anxiety, depression, PTSD, and pain after a four-day EFT training. Both in-person and virtual delivery of EFT produced statistically significant improvements in anxiety, depression, pain, and PTSD, with most improvements maintained over time. This finding is consistent with the literature demonstrating that online treatment is an effective method of delivering psychological therapies. We identified a greater percent change in symptoms in the in-person group. However, this data was collected pre-COVID while data collection for the virtual group occurred post-COVID, set in the context of a large global rise in anxiety and depression. RCTs and comparisons between in-person and virtual application performed post-covid are required to determine relative efficacy, as well as to test the theoretical underpinnings of EFT and develop consensus treatment guidelines. Virtual treatment improves patient access and group therapy lowers costs; thus, online EFT has the potential to play a role in the delivery of evidence-based mental health interventions. This study demonstrates that virtual EFT is effective for the most common psychological symptoms and, like in-person EFT, may be regarded as a front-line mental health treatment in primary care settings.

## Figures and Tables

**Table 1:** Baseline Characteristics of Participants in the Face-to-Face Delivery Compared to Virtual Delivery at Recruitment.

	Face-to-Face			Virtual			*p*
Demographic and Baseline Characteristics	Subjects	Male	Female	Subjects	Male	Female	
Gender (%)	203	33 (16.3)	170 (83.7)	172	19 (11.1)	153 (88.9)	0.435
Age, years							0.004 *
Mean	50.45	48.13	50.80	46.97	49.63	46.64	
Standard deviation	12.35	14.76	11.96	10.02	11.84	9.76	
Min–Max	19–81	22–75	19–81	23–79	30–73	23–79	
Education, *n* (%)							
High school/College	14 (6.9)	3 (9.09)	11 (6.47)	26 (15.02)	0 (0)	26 (17.0)	
University	55 (27.1)	9 (27.27)	46 (27.06)	111 (64.16)	11 (57.90)	100 (65.40)	
Postgraduate	86 (42.4)	15 (45.45)	71 (41.46)	35 (20.23)	8 (42.10)	27 (17.6)	
Unknown	48 (23.6)	6 (18.18)	42 (24.71)	0	0 (0)	0 (0)	
Post-traumatic stress symptoms							0.012 *
Mean	2.54	2.57	2.51	2.05	2.00	2.06	
Standard deviation	1.24	1.50	1.17	0.82	0.75	0.83	
Min–Max	1–5	1–5	1–5	1–5	1–3	1–5	
Pain							<0.001 *
Mean	4.09	3.54	4.16	3.04	2.84	3.06	
Standard deviation	2.49	2.40	2.49	2.38	2.24	2.40	
Min–Max	0–10	0–8	0–10	0–8	0–7	0–8	
Happiness							0.001 *
Mean	7.28	7.39	7.30	6.73	6.42	6.77	
Standard deviation	2.10	2.41	2.03	1.88	2.06	1.86	
Min–Max	0–10	0–10	1–10	2–10	2–10	2–10	
Anxiety	HADS			PHQ			--- **
Mean	8.35	7.31	8.54	1.64	1.53	1.65	
Standard deviation	3.85	3.95	3.82	1.32	1.12	1.34	
Min–Max	0–20	1–18	0–20	0–6	0–4	0–6	
Depression	HADS			PHQ			--- **
Mean	4.04	4.14	3.96	0.90	0.79	0.91	
Standard deviation	3.18	3.29	3.07	1.07	0.98	1.08	
Min–Max	0–14	0–13	0–13	0–6	0–3	0–6	

Note: * Significant difference. ** No *p*-value; as different scales were used, no statistical analysis could be carried out.

**Table 2:** Virtual Participant Outcome Measures Pre- Versus Post-intervention.

Scale	Pretest, Mean ± SD	Post-Test, Mean ± SD	Change in Mean	Z Statistic	*p*	Percent Change
Happiness (*n* = 172)	6.733 ± 1.876	7.826 ± 1.561	1.093	−7.092	<0.001	16.23
Anxiety (*n* = 172)	1.640 ± 1.315	1.256 ± 1.221	−0.384	3.987	<0.001	−23.41
Depression (*n* = 172)	0.895 ± 1.065	0.610 ± 0.861	−0.285	3.505	<0.001	−31.84
PTSD (*n* = 172)	4.006 ± 1.362	3.529 ± 1.550	−0.477	4.071	<0.001	−11.91
Pain (*n* = 172)	3.035 ± 2.377	1.936 ± 2.000	1.099	5.918	<0.001	−36.21

**Table 3:** Virtual Participant Outcome Measures Pre to 6-Month Follow-up.

Scale	Pretest, Mean ± SD (*n* = 172)	Follow-Up Mean ± SD (*n* = 92)	Change in Mean	*Z* Statistic	*p*	Percentage Change
Happiness (*n* = 92)	6.733 ± 1.876	7.413 ± 1.749	0.68	−3.491	<0.001	8.61
PHQ Anxiety (*n* = 92)	1.640 ± 1.315	1.446 ± 1.252	−0.194	1.588	0.102	−11.83
PHQ Depression (*n* = 92)	0.895 ± 1.065	0.674 ± 0.962	−0.221	1.875	0.048	−24.69
PSTD (*n* = 92)	4.006 ± 1.362	3.598 ± 1.490	−0.408	3.483	<0.001	−10.18
Pain (*n* = 92)	3.035 ± 2.377	2.315 ± 2.301	−0.72	3.038	0.002	−23.72

**Table 4:** Participant Outcome Measures Pre- Versus Post-intervention for Face-to-Face Compared to Virtual EFT.

	Face-to-Face (*n* = 170)			Virtual (*n* = 172)		
Scale	Change in Mean	*p*	Percent Change	Change in Mean	*p*	Percent Change
Happiness	1.37	<0.000	18.82	1.09	<0.001	16.23
Anxiety	−3.37	<0.000	−40.36	−0.384	<0.001	−23.41
Depression	−1.74	<0.000	−43.07	−0.29	<0.001	−31.84
PTSD	−1.44	<0.000	−30.38	−0.48	<0.001	−11.91
Pain	−2.46	<0.000	−60.15	1.10	<0.001	−36.21

**Table 5:** Participant Outcome Measures Pre- Versus Follow-up Intervention for Face-to-Face Compared to Virtual EFT.

	Face-to-Face (*n* = 85)			Virtual (*n* = 92)		
Scale	Change in Mean	*p*	Percent Change	Change in Mean	*p*	Percent Change
Happiness	0.42	0.114	5.77	0.68	<0.001	8.61
Anxiety	−2.73	<0.001	−32.69	−0.194	0.102	−11.83
Depression	−1.26	0.002	−31.18	−0.221	0.048	−24.69
PTSD	−1.10	<0.001	−23.21	−0.408	<0.001	−10.18
Pain	−1.43	<0.001	−34.96	−0.72	0.002	−23.72

**Table 6:** Comparison of Face-to-Face Versus Online Over Time.

	Face-to-Face Mean (SD)	Virtual Mean (SD)	Mann-Whitney U *p*-Value
**PTSD Symptoms**			
PCL Pre EFT	4.735 (2.155)	4.006 (1.362)	0.012 *
PCL Post EFT	3.303 (1.487)	4.855 (1.375)	<0.001 *
PCL Follow-up	3.642 (1.734)	3.589 (1.476)	0.826
**Happiness**			
Happiness pre-EFT	7.280 (2.098)	6.733 (1.876)	0.001 *
Happiness post	8.846 (1.715)	7.826 (1.561)	<0.001 *
Happiness Follow-up	7.698 (2.098)	7.413 (1.749)	0.069
**Pain**			
Pain Pre-EFT	4.093 (2.489)	3.035 (2.377)	<0.001 *
Pain Post-EFT	1.629 (1.889)	1.936 (2.000)	0.096
Pain follow-up	2.656 (2.261)	2.315 (2.301)	0.185

Note: * Statistically significant.

## Data Availability

The raw data supporting the conclusions of this article will be made available by the authors on request.
